# Does red blood cell irradiation and/or anemia trigger intestinal injury in premature infants with birth weight ≤ 1250 g? An observational birth cohort study

**DOI:** 10.1186/s12887-018-1241-5

**Published:** 2018-08-11

**Authors:** Terri Marin, Ravi M. Patel, John D. Roback, Sean R. Stowell, Ying Guo, Kirk Easley, Megan Warnock, Jane Skvarich, Cassandra D. Josephson

**Affiliations:** 1 Department of Physiological and Technological Nursing, Augusta University, College of Nursing, 1120 15th Street, EC-5354, Augusta, GA 30912 USA; 2 Department of Pediatrics, Emory University, School of Medicine, 2015 Uppergate Drive, Atlanta, GA 30322 USA; 3 Department of Pathology and Laboratory Medicine, Emory University, School of Medicine, 1364 Clifton Rd NE, Atlanta, GA 30322 USA; 4 Department of Biostatistics and Bioinformatics, Emory University, School of Public Health, 1518 Clifton Rd, Atlanta, GA 30322 USA

## Abstract

**Background:**

Necrotizing enterocolitis (NEC) is a leading cause of neonatal morbidity and mortality in premature infants. To date, no effective biomarkers exist to predict which premature infants will develop NEC, limiting targeted prevention strategies. Multiple observational studies have reported an association between the exposure to red blood cell (RBC) transfusion and/or anemia and the subsequent development of NEC; however, the underlying physiologic mechanisms of how these factors are independently associated with NEC remain unknown.

**Methods:**

In this paper, we outline our prospective, multicenter observational cohort study of infants with a birth weight ≤ 1250 g to investigate the associations between RBC transfusion, anemia, intestinal oxygenation and injury that lead to NEC. Our overarching hypothesis is that irradiation of RBC units followed by longer storage perturbs donor RBC metabolism and function, and these derangements are associated with paradoxical microvascular vasoconstriction and intestinal tissue hypoxia increasing the risk for injury and/or NEC in transfused premature infants with already impaired intestinal oxygenation due to significant anemia. To evaluate these associations, we are examining the relationship between prolonged irradiation storage time (pIST), RBC metabolomic profiles, and anemia on intestinal oxygenation non-invasively measured by near-infrared spectroscopy (NIRS), and the development of NEC in transfused premature infants.

**Discussion:**

Our study will address a critical scientific gap as to whether transfused RBC characteristics, such as irradiation and metabolism, impair intestinal function and/or microvascular circulation. Given the multifactorial etiology of NEC, preventative efforts will be more successful if clinicians understand the underlying pathophysiologic mechanisms and modifiable risk factors influencing the disease.

**Trial registration:**

Our study is registered in ClinicalTrials.gov Identifier: NCT02741648.

## Background

Necrotizing enterocolitis (NEC) is the most common gastrointestinal emergency among premature infants [1, 2], occurring in approximately 11% of those born < 29 weeks’ gestation [3]. Case-fatality rates are as high as 50% for extremely low birth weight (ELBW) infants (≤ 1000 g at birth) who develop NEC [4]. Survivors are at risk for substantial long-term complications including neurodevelopmental delay, nutritional deficit and failure to thrive [3, 5]. Costs associated with NEC in the United States are estimated at $1 billion annually [2]. Transfusion-related necrotizing enterocolitis (TR-NEC) refers to an observed phenomenon that specifically describes a premature infant who develops NEC within 48 h after receiving a red blood cell (RBC) transfusion. Several reports have identified RBC transfusions as a significant and independent risk factor for NEC [6–14]; however, others have not found an association [15–21], but rather an association with degree of anemia prior to NEC development, which has led to considerable controversy [18].

No current biomarkers reliably predict NEC, limiting efforts to prevent this disease. The range of symptoms are highly variable, from subtle signs such as feeding intolerance and abdominal distention, to complete cardiovascular collapse and shock. Because NEC can progress to extensive bowel necrosis within hours, therapies are often ineffective [22]. Multiple factors are related to NEC etiology including prematurity, enteral feeding, pro-inflammatory propensity of the immature intestine, and impaired mesenteric blood flow [23]. The majority of premature infants receive transfusions for anemia of prematurity, and RBC transfusions precede approximately 25–38% of NEC cases [7, 14, 24]. Transfusion of different storage aged RBCs to premature infants has not been shown to contribute to the risk of NEC [7, 24]. However, the chronological storage age of RBCs may not be an accurate gauge of donor RBC function and the storage lesion may be exacerbated by gamma irradiation [25], which is performed to prevent transfusion-associated graft-vs-host disease. Although the Age of Red Blood Cells in Premature Infants (ARIPI) trial investigated the effects of total storage duration of RBCs in preterm infants [26], the study did not investigate the effects of irradiation [27]. Currently, the “safe” duration of RBC storage following irradiation (post-irradiation storage time, pIST) is unclear. Given the multifactorial etiology of NEC, preventative efforts will be more successful if clinicians understand the underlying pathophysiologic mechanisms and modifiable risk factors influencing the disease.

Although premature infants weighting ≤1250 g at birth are frequently transfused for anemia of prematurity, optimal transfusion guidelines are ill-defined [28]. The Premature Infants in Need of Transfusion (PINT) trial [29] and the smaller Iowa trial [30] investigated the effects of transfusion practices on morbidity, although neither trial included NEC as the primary outcome. Because the PINT trial suggested lower hemoglobin thresholds decreased the number of RBC transfusions with no adverse effect on mortality, retinopathy of prematurity, or neurologic injury [29], many centers shifted to conservative transfusion practices. Concurrently, multiple published reports have described an association between RBC transfusion and NEC [6, 7, 10, 24, 31–33], although meta-analyses have shown conflciting findings regarding any association [11, 34]. The lack of adequately-powered randomized trials evaluating the effect of transfusion thresholds on NEC limit determination of whether increased tolerance of neonatal anemia by use of conservative transfusion thresholds may actually increase the risk of NEC.

A causal link between RBC transfusion and NEC has been proposed, but not proven. Our previous research described a matched case-control study of 184 very low birth weight (VLBW) infants weighing ≤1500 g with NEC, and found a higher risk of late-onset NEC (after 4 weeks of age) in transfused infants (OR 6.7; 95% CI: 1.5–31.2) [8]. An initial meta-analysis of observational studies also showed increased risk of NEC in VLBW transfused infants [11], although a more-recent meta-analysis found no association [34] with findings consistent from our recent multicenter, prospective cohort study [18]. Many of the studies included in the meta-analyses [34] were observational and limited in causal inference; no studies have provided data regarding the potential underlying pathophysiologic mechanisms. While these studies identified risk factors for NEC, including severity of anemia and a developmental window at which NEC occurs, few studies have focused on characteristics of the donor RBC transfusion, such as pIST and metabolic/functional abnormalities. Therefore, the critical scientific gap that remains to be addressed is whether transfused RBC characteristics, such as irradiation and metabolism, impair intestinal function and/or microvascular circulation. Our current investigation aims to prospectively evaluate the relationship between pIST, RBC metabolomic profiles, and anemia on mesenteric oxygenation, as measured by near-infrared spectroscopy (NIRS), and NEC.

### Candidate biological mechanisms of NEC

A number of potential mechanisms and clinical factors with biologic plausibility support a potential causal connection between RBC transfusion in response to anemia and NEC, despite the limitations described previously. Underlying this association is a common central component of insufficient oxygen delivery to intestinal tissue from a combination of decreased oxygen carrying capacity (anemia) and/or decreased blood flow (cardiac output, vascular tone). Oxygen consumption and extraction in intestinal tissue beds can be continuously and non-invasively monitored by near-infrared spectroscopy (NIRS) through measurement of oxygenated versus deoxygenated hemoglobin in venous (75%) and capillary (25%) blood [35].

#### Severity of Anemia and oxygen delivery to intestines

Severe anemia, leading to decreased oxygen delivery, may cause intestinal injury that predisposes an infant to NEC. Alkalay and colleagues [36] demonstrated that infants who appeared clinically “stable” with either significant anemia (hematocrit < 21%) or milder anemia (hematocrit 22–26%) had high cardiac output and restricted intestinal blood flow. Singh calculated that each percent decrease in nadir hematocrit led to a 10% increase in odds for NEC (OR 1.10; 95% CI: 1.02–1.18; *P* = 0.01) [10]. In our retrospective study, infants who developed NEC after transfusion had lower hematocrits 1 week prior than those without NEC [8]. We also found lower mesenteric oxygen saturation (MES-rSO_2_) measured by NIRS during and after transfusions in infants who developed NEC, and enteral feedings given during RBC transfusion worsened this effect [9]. Furthermore, a recent prospective study from our group found the rate of NEC was significantly increased among VLBW infants with severe anemia (≤ 8 g/dL) in a given week compared with those who did not have severe anemia (adjusted cause-specific hazard ratio, 5.99 [95% CI, 2.00–18.0]; *p* = .001) [18]. However, no study to date has prospectively compared longitudinal hemoglobin/hematocrit measures, MES-rSO_2_, and development of NEC in this population.

#### The RBC storage lesion, irradiation, nitric oxide (NO), and NEC

RBCs mediate local blood flow to preferentially perfuse the most hypoxic tissues, a process termed hypoxic vasodilation [36]. Nitric oxide (NO) released by RBCs is a potential mediator [37]. However, RBCs can also scavenge NO, a vasoconstrictive activity that may be enhanced in transfused RBCs with longer storage [38, 39]. Therefore, transfusion of stored RBCs (“storage-aged RBCs”, saRBCs) or pre-storage irradiated saRBCs (which worsens storage lesion) [40, 41] may disrupt vascular tone and blood flow. In animal studies, blood vessels of the immature intestine vasoconstrict when NO is depleted [42–45]. Thus, in a preterm infant with anemia, NEC could result from two mechanisms: transfusion of saRBCs (including those with extended pIST) interacting with immature intestinal endothelium, which together synergistically reduce blood flow, causing tissue hypoxia and, in some cases, NEC.

##### Aims

**Fig. 1:**
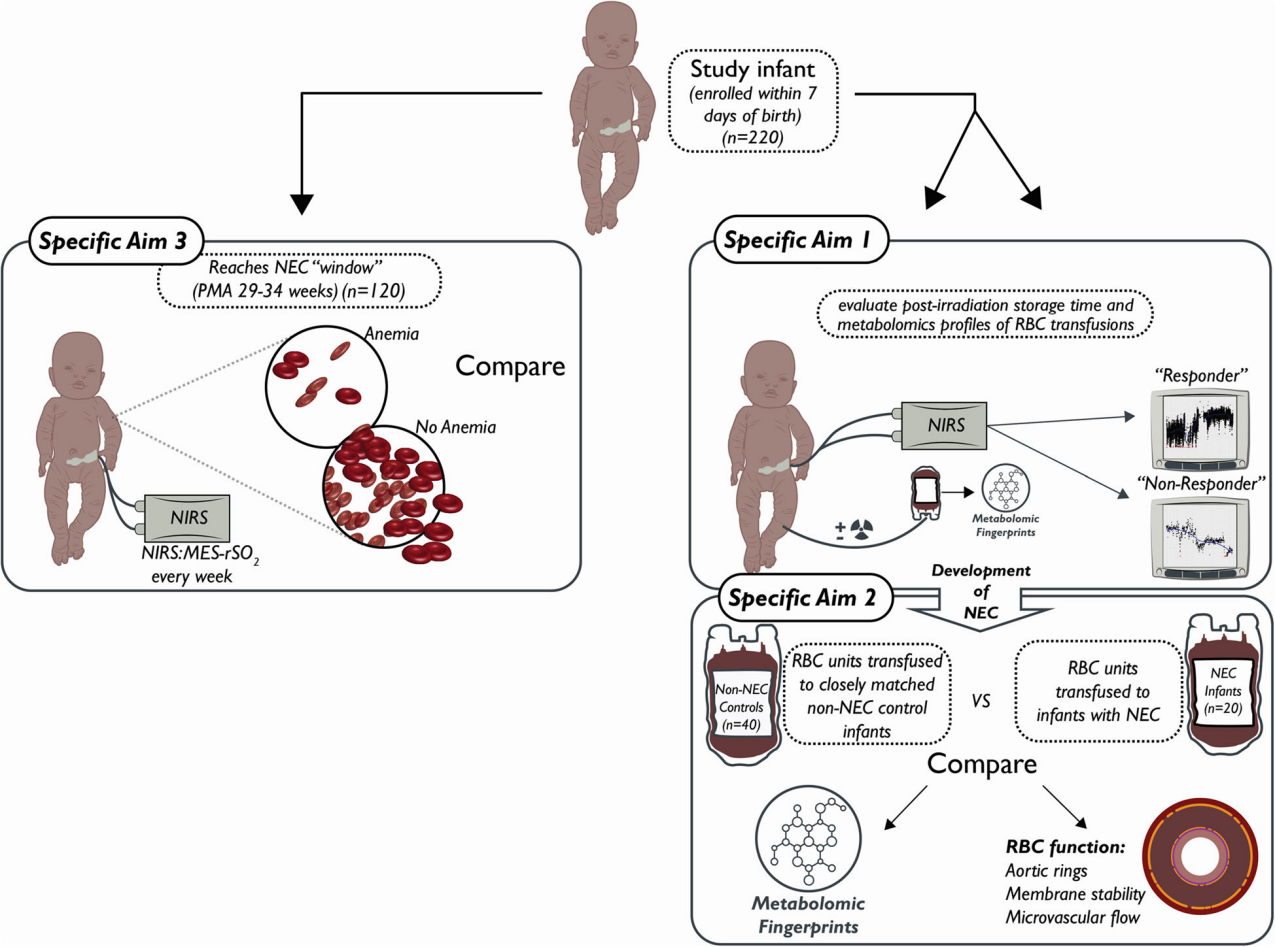
Study schema illustrating our specific aims, projected infant enrollment, and methodologic approach. Our prospective, observational cohort investigation will determine the associations of prolonged irradiation time (pIST) and metabolic changes of transfused RBCs to alterations in mesenteric oxygenation that may increase the risk for NEC in preterm infants weighing ≤1250 g. In addition, we will explore the implications of severe anemia (hemoglobin ≤8 g/dL) when infants are most vulnerable to NEC development, approximately 29–34 weeks’ gestation. Abbreviations: NIRS, near-infrared spectroscopy, RBC, red blood cell; PMA, post menstrual age; NEC, necrotizing enterocolitis; mesSO_2_, mesenteric regional oxygen saturation

The aims of this prospective, observational study are to (Fig. 1): 1) Determine the associations between RBC storage after irradiation (pIST), metabolic alterations in stored/irradiated RBCs, and changes in mesenteric digestive tract regional saturation of oxygen (MES-rSO_2_) measured by NIRS; 2) Compare in vitro measures of RBC function, and metabolic changes, between RBC products transfused to infants who develop NEC compared to matched control infants who do not; and 3) Explore the clinical implications of severe anemia (hemoglobin ≤8 g/dL) during the vulnerable “NEC window period” of 29–34 weeks postmenstrual age in the responses of infants < 1250 g to RBC transfusion. Our overarching hypothesis is that irradiation of RBC units followed by longer storage perturbs RBC metabolism and function leading to paradoxical microvascular constriction, mesenteric tissue hypoxia, and increased risk for NEC in transfused premature infants with already impaired intestinal oxygenation secondary to significant anemia.

##### Study design

We will prospectively investigate, using an established birth cohort design [18, 46], the associations between RBC transfusion (including characteristics of transfused RBCs), anemia, intestinal oxygenation, and NEC. We specifically aim to understand the relationship between pIST, donor RBC metabolomics profiles, and recipient anemia on MES-rSO_2_, as measured by NIRS, and the development of NEC (Fig. 1). Although many studies have characterized the association between NEC and transfusion, none have focused on improving our understanding of the underlying pathophysiology, particularly the intestinal oxygenation changes preceding NEC. Further, the safety and efficacy of various blood banking practices including the preparation of storage-aged RBCs (saRBCs) and repeat donor exposure in recipients remains unclear. The safe threshold of saRBC+/− pIST in infants is not known and longer-stored saRBCs, either before or after irradiation, may potentiate the RBC storage lesion. This study may provide new knowledge regarding the potential benefit or harm of various blood banking practices and may identify new potential mediators of NEC.

The study schema (Fig. 1) illustrates how we aim to characterize the association between metabolic changes in transfused RBCs relative to pIST, and adverse effects in the recipient through two approaches: in vivo NIRS trend monitoring of intestinal oxygenation, and in vitro RBC functional studies. The schema explains how variables will be compared to evaluate primary and secondary endpoints for each specific aim. Our primary endpoint is changes in MES-rSO_2_ trends in response to transfusion of saRBC+/− pIST as measured by NIRS (Aim 1). Secondary endpoints are 1) to determine the hazard ratio of NEC comparing infants transfused with saRBCs with and without pIST; (2) examination of metabolomics fingerprints of transfused RBC (in vitro) among infants with and without NEC (Aim 2); and 3) impact of severity of anemia over time during the NEC window (29–34 weeks postmenstrual age) on MES-rSO_2_ (Aim 3).

##### Study population and eligibility criteria

Our Emory University Institutional Board Review approved study we will enroll subjects at three sites in metro-Atlanta, Georgia. All infants with birthweight ≤1250 g in any of the 3 participating neonatal intensive care will be eligible for enrollment. Infants to be excluded are those who are not expected to live beyond 7 days of life (based on assessment of attending neonatologist), presence of a severe congenital anomaly, RBC or platelet transfusion received at an outside facility or prior to study screening, or maternal refusal to participate. Written informed consent from a parent or guardian will be obtained by a study investigator for each patient before enrollment. Infants will be screened for eligibility, and enrolled within 7 days of birth.

##### Sample size estimation

We aim to enroll a total of 220 infants into the study. We assume 110 of these infants (50%) will receive RBC transfusion and undergo NIRS monitoring. Analysis comparing two groups, divided equally among infants by pIST, would provide more than 80% power to detect a difference of 10% in pre- and post-transfusion mean area under the curve MES-rSO_2_ change (standard deviation = 18) between groups or 90% power to detect a difference as small as 6% in MES-rSO_2_ with a standard deviation of 9 (Fig. 1 and Table 1). However, the number of infants enrolled in the study that receive a transfusion and have NIRS monitoring may be lower than 110. Therefore, we have provided power estimates for analysis of a sample size of 72 infants with RBC transfusion and NIRS monitoring (33% of the enrolled cohort). This will generate 80% power to detect a difference of 12% in pre- and post-transfusion mean area under the curve MES-rSO_2_ change between the two groups if the final sample size is 72 infants (36 per group) and the estimated standard deviation for MES-rSO_2_ change is 18. A secondary analysis evaluating pIST a continuous variable will also be performed, which will likely yield greater power for each of the scenarios presented.

**Table 1:** Sample Size and Power Estimates

Groups	*n* = 220 transfused				*n* = 110 transfused				*n* = 72 transfused			
	SD = 9		SD = 18		SD = 9		SD = 18		SD = 9		SD = 18	
Difference in MES-rSO_2_ AUC change (%)	Effect Size	Power	Effect Size	Power	Effect Size	Power	Effect Size	Power	Effect Size	Power	Effect Size	Power
6	0.67	1	0.33	0.69	0.67	0.93	0.33	0.41				
8	0.89	1	0.44	0.91	0.89	1	0.44	0.64				
10	1.11	1	0.56	0.98	1.11	1	0.56	0.82	1.11	1	0.56	0.64
12	1.33	1	0.67	1	1.33	1	0.67	0.93	1.33	1	0.67	0.8

Abbreviations: *MES-rSO*_*2*_ mesenteric regional oxygen saturation, *AUC* area under curve, *SD* standard deviation

## Methods

All RBC transfusions given to infants during hospitalization will be studied. All RBC transfused units are stored in citrate-phosphate-dextrose-adenine (CPDA-1) preservative solution. pIST and storage days will be recorded for each RBC transfusion. All infants will be monitored with NIRS prior to, during and up to 48 h following each transfusion. Consistent with epidemiologic reports of transfusion-related NEC and prior studies at the 3 centers, we anticipate approximately 20 (10%) infants will develop NEC while on study. For our 2:1 case-controlled metabolomic analysis, we will prospectively analyze 40 infants who do not develop NEC and compare to 20 infants with NEC. Within this sub-cohort, we will compare alterations in metabolic pathways from saRBC unit (in vitro) and infant blood sample (in vivo). We will then examine a third sub-cohort of 120 infants without NEC within the NEC “window” (29–34 postmenstrual weeks’). These infants will also be monitored weekly for 24–48 h with mesenteric NIRS to evaluate the relationship between anemic (hemoglobin < 8 g/dL) and non-anemic infants. Reports suggest that this specific population of infants are more likely to experience paradoxical reductions in MES-rSO_2_ substantially increasing the risk for NEC when transfusions are given [10, 36]. Analysis will also include assessment of hemoglobin as a continuous variable.

### Data management and quality control

To ensure data quality and procedural adherence of our statistical analysis approach, we will implement a detailed data management plan. Quality control will be applied to each phase of data handling to safeguard data collection and process reliability.

#### Birth cohort data

Case report form data, as defined and dictated by our study protocol, will be collected and managed using iDataFax, an electronic data capture application with extensive management features including a data query system to help ensure study credibility. The iDataFax system will generate regular reports that summarize and track routine data collection. These reports will help the investigative team monitor and maintain data completeness during follow-up and achieve high data capture performance by minimizing missed scheduled clinical assessments, preventing or reducing missing data, and maintaining high cohort retention rates over the three months of regular infant assessment at our three participating centers.

#### NIRS data

NIRS data will be downloaded daily and uploaded to a secure server within 24 h of monitoring completion. The data coordinating center will download and process all NIRS files on a weekly basis. During data processing, quality control reports will be generated to summarize the expected and actual duration of NIRS monitoring, percent of missing data, and identify when 30 min or more of consecutive data are missing. This approach will ensure proper data collection for future analysis for the entire duration of the monitoring period and confirm that NIRS machines are working properly. If one of our checks fails, we will notify the study nurses who will flag the machine, assess and correct the issue. If issues continue, we will notify the NIRS machine manufacturer, Medtronic, Inc. (Boulder, CO) for technical support. These steps will ensure consistency of data collection across our three study sites. In addition to individual NIRS monitoring checks, quarterly reports summarizing the total number of patients with NIRS monitoring, patient characteristics, and summary statistics for measurements collected during NIRS monitoring will be generated and reviewed by study investigators and biostatisticians.

### Primary outcome

All infants enrolled who receive RBC transfusion will have MES-rSO_2_ measured by NIRS as the primary study end-point [INVOS 5100C Cerebral/Somatic Oximeter (Covidien, Boulder, CO)], a Food and Drug Administration approved device for use on premature infants. NIRS noninvasively measures regional tissue saturation (rSO_2_) in real time because it calculates the difference between oxyhemoglobin (HbO_2_) and deoxyhemoglobin (HHb) expressed as: rSO_2_ = HbO_2_/HbO_2_ + HHb [47]. WE will obtain a baseline measurement by placing the NIRS probes on the infant at least 30 min prior to transfusion (triggered by the decision to transfuse made by the clinical team). Probes will remain in place to collect data for 48 h following transfusion completion. Two-probe site monitoring on mesenteric and renal beds will be used to evaluate differential tissue bed oxygenation. Adhesive sensor probes are vertically applied to left periumbilical area for mesenteric monitoring and horizontally to right flank for renal monitoring.

### Secondary outcomes

We will examine the association between metabolic features of transfused RBC units and pre-transfusion pIST, alterations in MES-rSO_2_, and the development of NEC. Our analysis will include methods previously used [25, 48]. Our preliminary data examining distinct effects of gamma irradiation on saRBC identified four metabolite pathways that were significantly altered by storage (> 7 days) and irradiation: arachidonic acid, linoleic acid, steroid biosynthesis, and alpha-linoleic acid. Alterations in these pathways may worsen RBC function, and we propose this may be involved with adverse intestinal oxygenation following RBC transfusion that, in some infants, could lead to NEC. However, we will not pre-select pathways for the current analysis. Therefore, we will pursue analytic approaches previously described [25] to identify metabolites that discriminate those infants with paradoxical MES-rSO_2_ responses with NEC to unaffected infants, and we will also conduct an additional secondary analyses focused on biochemical pathways previously identified in storage saRBCs generated from metabolomics analyses.

### Statistical methods

We will use a novel statistical approach [51] implemented by the *NIRStat* R package for analyzing the NIRS data. Specifically, the *NIRStat* method models the observed MES-rSO_2_ time series with a nonparametric smooth function via penalized regression splines [49, 50]. It then provides accurate and robust statistical measures for characterizing the important features in rSO_2_ series. We will use the mean area under the fitted spline curves (MAUC) measure generated from the *NIRStat* package to measure the MES-rSO_2_ levels at baseline and then at post-transfusion. The MAUC changes from baseline to post-transfusion will be used to quantify the changes in MES-rSO_2_ due to transfusion. Two sample t-tests will be used to compare the changes in MAUC between saRBCs +/− pIST. Multivariate linear regression models will also be applied to model the changes in AUC in terms of pIST status (with pIST and without pIST) and other potential confounding factors. The multivariate analysis will allow us to assess whether the changes in MES-rSO_2_ differs significantly between saRBCs with pIST and those without pIST, controlling for other confounding factors.

The incidence of NEC and death will be estimated by the cumulative incidence function appropriate for competing risks. Gray’s method (modified log-rank test) will be used to compare NEC cumulative incidence according to baseline clinical characteristics. Cause-specific hazard ratios will be calculated to measure the degree of association between baseline characteristics and NEC, and between baseline characteristics and death by fitting a stratified Cox proportional-hazards regression model for competing risks. The competing risks model will be implemented using SAS PHREG using robust sandwich covariance matrix estimates to account for within-mother correlation that may occur in outcomes of multiple-birth infants.

To guard against model overfitting, we will employ both clinical and statistical criteria in making decisions about which independent variables to include; and we will limit the number of candidate variables. In general, the results of models having fewer than 10 outcome events per independent variable are thought to have questionable accuracy and the tests of statistical significance may be invalid. The use of “machine-learning” covariate selection methods, such as bootstrap bagging, will be utilized to improve the reliability of identifying risk factors for NEC and death. The hazard ratio and its 95% confidence interval (CI) will be calculated for each factor in the presence of others in the final model for NEC and mortality.

For metabolomics analysis, we will examine associations between metabolic features of transfused RBC units and pre-transfusion pIST, alterations in MES-rSO_2_, and the development of NEC using an approach previously described [25]. Correlative analyses without pre-selecting specific metabolic pathways will be performed. Methods of analysis will include a number of gene set analysis, such as MSEA and MetaboAnalyst. We can also borrow from the gene expression packages to conduct more complex analysis of metabolite-set differential expression analysis as well as metabolite-set differential coordination analyses. The pathway level analysis will be followed by the detection of metabolites that contribute the most to the changes of metabolic pattern using the built-in scoring system of the packages.

## Discussion

There is an urgent need for a large, hypothesis-driven, prospective study to examine the effect of both RBC unit and recipient factors on the physiologic perturbations that cause NEC [11]. Given that 75–90% of low birth weight infants receive one or more RBC transfusions [52, 53], it is reasonable to predict that NEC may result from a combination of pre-existing anemia and reduced intestinal oxygenation exacerbated by metabolic/functional changes in transfused RBCs, due to irradiation and pIST. This investigation may allow us to identify new modifiable factors that can be used to test targeted prevention strategies and mitigate this devastating disease.

We propose to investigate intestinal oxygenation changes that precede the development of NEC. Our overarching hypothesis is that irradiation of RBC units followed by longer storage times perturbs donor RBC metabolism and function, and these derangements are associated with paradoxical microvascular vasoconstriction, intestinal tissue hypoxia and injury and/or NEC in transfused premature infants with already impaired intestinal oxygenation due to significant anemia. Specifically, our primary goal is to characterize the association between metabolic changes in transfused RBCs relative to pIST, and adverse effects in the recipient by in vivo NIRS trend monitoring of intestinal oxygenation, and in vitro RBC functional studies. Our primary endpoint is changes in MES-rSO_2_ trends in response to transfusion of saRBC+/-pIST as measured by NIRS. The secondary endpoints are to determine the hazard ratio of NEC for low birth weight infants transfused with saRBCs +/-pIST, examine metabolomic fingerprints of transfused RBC in vitro, and examine the impact of anemia severity on MES-rSO_2_ trends.

## Abbreviations

CI: Confidence interval
CPDA-1: Citrate-phosphate-dextrose-adenine
ELBW: Extremely low birth weight
HbO_2_: Oxyhemoglobin
HHb: Deoxyhemoglobin
MAUC: Mean area under the curve
MES-rSO_2_: Mesenteric regional oxygen saturation
NEC: Necrotizing enterocolitis
NIRS: Near-infrared spectroscopy
NO: Nitric oxide
OR: Odds ratio
pIST: prolonged irradiation storage time
RBC: Red blood cell
rSO_2_: regional oxygen saturation
saRBCs: storage-aged red blood cells
TR-NEC: Transfusion-related necrotizing enterocolitis
VLBW: Very low birth weight
